# Dynamic changes in excitability and viability of sporadic and *SOD1*-related amyotrophic lateral sclerosis iPSC-derived motor neurons

**DOI:** 10.3389/fcell.2026.1755814

**Published:** 2026-03-30

**Authors:** Ming Qi, Nan Hu, Jianfeng Ding, Jingwen Niu, Bo Long, Mingsheng Liu

**Affiliations:** 1 Department of Neurology, Peking Union Medical College Hospital, Chinese Academy of Medical Sciences and Peking Union Medical College, Beijing, China; 2 State Key Laboratory of Complex Severe and Rare Diseases, Peking Union Medical College Hospital, Peking Union Medical College and Chinese Academy of Medical Sciences, Beijing, China

**Keywords:** ALS, apoptosis, hyperexcitability, IPSC, motor neuron (MN)

## Abstract

**Objective:**

To explore the dynamic changes in excitability and viability of induced pluripotent stem cells (iPSC)-derived motor neurons from sporadic amyotrophic lateral sclerosis (ALS) and compare them with *SOD1*-related ALS patients and healthy control.

**Methods:**

Peripheral blood samples were collected from ALS patients and healthy controls (HC) to establish the iPSC-derived motor neurons (MNs). Whole-cell patch-clamp recordings at different culture stages was made using an Axopatch 700B amplifier in combination with pClamp 11 software (Molecular Devices). The frequency of action potentials (APs) was recorded. Additionally, Terminal deoxynucleotidyl transferase (TdT)-mediated deoxyuridine triphosphate (dUTP) Nick-End Labeling (TUNEL) was used to assess the apoptosis of MNs.

**Results:**

ALS patient-derived MNs exhibited significantly higher firing rates compared to HCs at both 4–7 weeks (p = 0.004) and 7–9 weeks (p = 0.009). Further analysis revealed that SOD1-derived MNs showed significantly higher firing frequencies than sALS (p = 0.009) and HCs (p < 0.001) in 4–7 weeks. In 7–9 weeks, it remained significant between SOD1 and HC-derived MNs (p = 0.015), but became insignificant between SOD1 and sALS (p = 0.855). The apoptotic rate of sALS (Day 30: 61.37% ± 9.63%; Day 60: 78.41% ± 6.63%) and SOD1 (Day 30: 73.69% ± 8.81%; Day 60: 60.37% ± 11.53%) -derived MNs was significantly higher than those of HCs at both Day 30 (30.72% ± 7.57%) and Day 60 (50.85% ± 19.36%) (p < 0.001).

**Conclusion:**

MNs derived from both patients with mutant SOD1 and sporadic ALS exhibited increased excitability compared to HCs. The increased excitability of MNs derived from ALS patients with mutant SOD1 occurred earlier, and over time, became consistent with the excitability observed in MNs derived from sporadic ALS. The apoptosis rates of MNs showed similar trends. iPSC-derived MNs from both sporadic and mutant ALS may serve as useful cell models for ALS in future studies.

## Graphical Abstract

**Figure:**
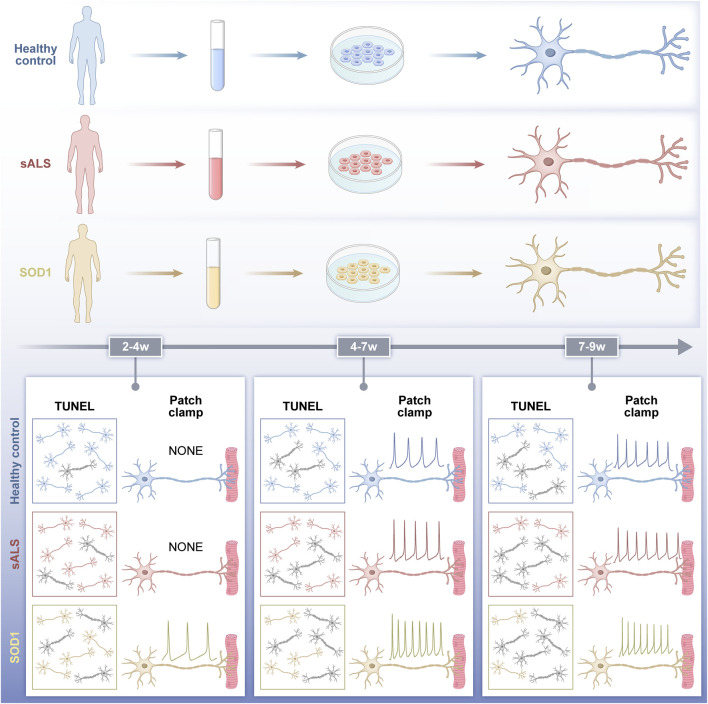

## 1 Introduction

Amyotrophic lateral sclerosis (ALS) is a rare neurodegenerative disorder that affects both upper and lower motor neuron (UMN and LMN), resulting in incurable muscle weakness and atrophy. The pathogenesis of ALS remains unclear. Genetic testing indicates that 5%–10% of ALS cases are associated with gene mutations, including mutant *SOD1*, *C9ORF72*, *TARDBP*, and *FUS*, which contribute to familial ALS (Al-Chalabi and Hardiman, 2013). These genes and their mutant proteins may facilitate ALS onset through distinct pathological pathways. In recent years, significant advances has been made in drug development for familial ALS. For instance, Tofersen, targeting *SOD1*-related ALS, has been shown to markedly reduce neurofilament light chain (Nfl) levels in cerebrospinal fluid (CSF), though its impact on clinical manifestations requires further investigation (Miller et al., 2022). The majority of ALS cases, however, are sporadic, lacking a family history or identifiable pathogenic mutations in whole-genome sequencing (WES). Research on the mechanisms and drug development for sporadic ALS is particularly challenging due to the absence of clear genetic targets.

Abnormal motor neuron excitability plays a crucial role in ALS pathogenesis. Ultrasonographic studies have revealed widespread and frequent fasciculations in ALS patients, which serve as clinical indicators of motor neuron hyperexcitability (Swash and Carvalho, 2011; Liu et al., 2021). These fasciculations may emerge prior to muscle weakness and precede electromyographic changes. As the disease progresses and muscle atrophy worsens, fasciculations gradually diminish. At the cellular level, most studies suggest that ALS is characterized by motor neuron hyperexcitability, evidenced by reduced threshold potentials, increased action potential firing frequencies, and membrane depolarization (Geevasinga et al., 2016). However, some studies propose that motor neurons in ALS may eventually enter a state of electrophysiological dysfunction, exhibiting abnormally reduced excitability (Geevasinga et al., 2016). Animal studies have demonstrated increased excitability in motor cortical neurons as early as the embryonic stage, which declines as the disease progresses, ultimately leading to neuronal death (Brunet et al., 2020). These findings suggest that abnormalities in motor neuron excitability in ALS begin early, persist throughout the disease course, and undergo dynamic changes. Investigating the characteristics and underlying mechanisms of motor neuron excitability dysfunction may provide insights into ALS pathogenesis and identify novel therapeutic targets.

The mechanisms underlying motor neuron excitability abnormalities in ALS remain unclear, but may involve ion channel dysfunction, neurotransmitter imbalances, and neuronal damage (Geevasinga et al., 2016). Patch-clamp electrophysiology, applied to cultured motor neurons, brain slices, or spinal cord slices, is an effective technique for assessing the functional states of various ion channels in ALS. Kuo et al. used patch-clamp techniques to study spinal cord slices from SOD1G93A mice at both embryonic and postnatal stages (cultured for 2–4 weeks). They observed increased motor neuron excitability without changes in cell morphology, size, resting membrane potential, or input resistance compared to wild-type mice (Kuo et al., 2004). In 2009, Karumbayaram et al. were the first to record action potentials in motor neurons derived from induced pluripotent stem cells (iPSCs) of ALS patients using patch-clamp techniques (Karumbayaram et al., 2009). Since then, iPSC-derived motor neurons have been widely adopted as models for studying ALS pathogenesis and drug screening (Nakamura et al., 2023; Workman et al., 2023). Compared to animal models, iPSC-derived motor neurons are useful for studying both familial and sporadic ALS, facilitating mechanistic and therapeutic research. Additionally, motor neurons from ALS patients may exhibit early morphological alterations, including synaptic loss, dendritic atrophy, and reduced spine density, which correlate with neurodegeneration and hyperexcitability (Fujimori et al., 2018). Immunohistochemical staining of iPSC-derived motor neurons for specific markers, membrane protein expression, and neurite outgrowth is a valuable method for assessing neuronal function and apoptosis, and is widely used in ALS drug testing (Fujimori et al., 2018).

In the study, we aim to explore the dynamic changes in excitability of iPSC-derived motor neurons from sporadic ALS and compare with *SOD1*-related ALS patients using patch-clamp techniques. Furthermore, immunofluorescence staining will be employed to examine apoptosis in ALS motor neurons at different cultivation stages. We hope to further elucidate the characteristics and mechanisms of motor neuron excitability dysfunction in ALS, establish an iPSC-based model for sporadic ALS research, and shed new lights for future studies.

## 2 Methods

### 2.1 Patients and materials

Patients diagnosed with definite or probable ALS according to the revised El Escorial criteria (Brooks et al., 2000) were recruited from Peking Union Medical College Hospital (PUMCH). Enrolled patients were documented for their name, gender, age, clinical symptoms, medications, and underwent a detailed physical examination. ALSFRS-R was recorded during clinical assessments.Peripheral blood was collected at the time of diagnosis (within 3 months of symptom onset) for both ALS patients. Next-generation sequencing (NGS) was performed to confirm genetic mutations in familial ALS (fALS) and to exclude genetic mutations in sporadic ALS (sALS). Controls were healthy individuals without a history of psychiatric or neurological disorders.

The study was approved by the Ethics Committee of the Peking Union Medical College Hospital (PUMCH) (JS1210). Informed consent was obtained from all participants and/or their legal guardians.

### 2.2 Establishment and culture of iPSC-derived motor neurons

Peripheral blood samples were collected from ALS patients and healthy controls (HCs) (30 years old, female) to obtain peripheral blood mononuclear cells (PBMCs). The establishment of iPSC-derived motor neurons (MNs) followed the reported protocol (thermofisher A16518, CytoTune™-iPS 2.0 Sendai Reprogramming Kit). As shown in Supplementary Figures 1, 2, human iPSC cells were successfully differentiated into early-stage MNs.

MNs derived from iPSCs were plated at a density of 1 × 10^5^ cells per well in a 24-well plate coated with growth-factor-reduced Matrigel (Corning). MNs from fALS, sALS, and HC were inoculated into three culture wells in the same column. Motor neurons were transfected daily with N-SA/O-SA mRNAs for 3 days. Culture medium with SHH (100 ng/mL) and DAPT (10 μM) were changed daily and shifted from mTeSR1(Stem Cell Technologies) to N2 (Thermo Fisher Scientific) in 3 days. Cells were dissociated by Accutase (Stem Cell Technologies) and replated to poly-D-Lysine/Laminin-coated surface at the density of 1 × 105 cells/cm^2^. Neuron maturation medium contained neurobasal medium with the B27 supplement, BDNF (10 ng/mL), GDNF (10 ng/mL), cAMP (0.1 mM), ascorbic acid (0.2 mM), DAPT (10 μM). Medium was changed after 48 h followed by half change every 3–4 days. Cryopreservation medium contained 40% neurobasal medium, 50% FBS and 10% DMSO (Supplementary Figure 3).

All chemicals were from Sigma Aldrich unless otherwise mentioned.

### 2.3 Excitability assessment of motor neurons using patch-clamp techniques

**FIGURE 1:**
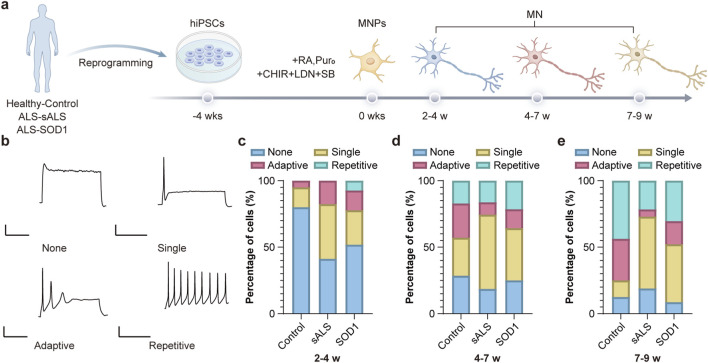
Evoked firing types of iPSC-derived MNs cultured for different time periods. **(a)** Schematic showing differentiation of iPSCs into MNs. **(b)** A Four types of evoked APs upon current stimulation. **(c–e)** Percentages of MNs derived from control (n = 71 cells), mutant SOD1 (n = 78 cells), and sALS (n = 97 cells) showing each type of firing cultured for 2–4 weeks **(c)** 4–7 weeks **(d)** and 7–9 weeks **(e)**.

Motor neurons (MNs) were plated onto coverslips and selected for electrophysiological analysis based on morphology. (Figure 1a) Neurons were chosen for patch clamping both within and outside neuronal clusters. Due to the enrichment of MNs in the culture, most of the recorded cells were MNs. Although live cell-type specific labeling was not performed, the possibility that some recordings may have originated from interneurons cannot be excluded. The spread of passive properties in the data did not suggest a separation between cell types. Cells with series resistance (Ra) greater than 40 MΩ were excluded, and recordings with changes in Ra or cell capacitance greater than 10% during the recording were omitted. Any cell lines stained with less than 30% MNs were excluded. Immunostaining for motor neuron markers (ISL1, HB9) at the time of recording indicated that ≥85% of cells in culture were MNs.

Internal solution (mM) used were: 140 Potassium Gluconate, 6 NaCl, 1 EGTA, 10 HEPES, 4 MgATP, 0.4 Na_3_GTP, adjusted to pH of 7.3 using KOH and osmolarity 290 mOsm. Artificial CSF included (mM): 167 NaCl, 2.4 KCl, 1 MgCl_2_, 10 Glucose, 10 HEPES and 2 CaCl_2_ adjusted to a final pH of 7.4 and osmolarity of 300 mOsm.

MNs were identified using a ×10 objective mounted on an upright microscope with transmitted light, and their neuronal soma were then visualized through a ×40 water immersion objective using IR differential interference contrast optics (DIC) (Figure 3a). The cell somatic recordings were made using an Axopatch 700B amplifier in combination with pClamp 11 software (Molecular Devices). Neurons were initially voltage-clamped at −70 mV, and Rseries and Rinput were monitored using a 0–200 pA step in each recording sweep. Next, recording was switched to current clamp. The resting membrane potential and the action potential (AP) were monitored for more than 5 min. To induce APs, the neurons were commanded by multiple steps of hyperpolarization currents. Electrophysiological recording data were first visualized with Clampfit 11 and exported to Graphpadprism for analysis.

For each AP, we recorded: (1) the frequency (Hz): the number of APs per second; (2) Threshold (mV): the minimum membrane potential required to trigger an AP; (3) Peak amplitude (mV): the magnitude of membrane potential change from threshold to peak; (4) Half-width (ms): the time duration corresponding to the decay of the AP from the peak to half-peak amplitude; (5) Max rise slope (V/ms): the maximum rate of depolarization during the AP upstroke; (6) After hyper polarization (AHP) (mV): the amplitude of hyper polarization potential after AP (Figure 3b).

### 2.4 Morphological and apoptotic rate assessment of motor neurons

Cells were fixed with 4% paraformaldehyde (PFA) for 30 min. After three washes with PBS, cells were permeabilized with 0.1% Triton X-100 in PBS for 30 min and then blocked with 5% bovine serum albumin (BSA) in DPBS for another 30 min. Subsequently, cells were incubated with the primary antibody overnight at 4 °C. After three additional washes with DPBS, cells were incubated with various fluorescently labeled secondary antibodies and Hoechst in blocking buffer for 1 h at room temperature. Antibodies used included OCT4 (Abcam), SOX2 (Abcam), NANOG (Abcam), SSEA4 (Abcam), CHAT (Sigma), TUJ1 (Stemcell), and ISL1 (Abcam).

For soma imaging spinal motor neurons were identified based on ISL1 marker area and nuclear morphology and imaged on a Nikon A1R confocal microscope. These images were analyzed using ImageJ software. Three independent experiments were conducted.

Terminal deoxynucleotidyl transferase (TdT)-mediated deoxyuridine triphosphate (dUTP) Nick-End Labeling (TUNEL) (Beyotime Biotechnology,C1086) was used to detect the apoptosis of MNs.

### 2.5 Statistics

Continuous variables are expressed as mean ± standard deviation (SD), while categorical variables are presented as counts and relevant percentages. Chi-square analysis was used to compare the frequencies of different types of evoked firing potentials. Independent t-tests or one-way analysis of variance (ANOVA) were used for comparisons of electrophysiological variables between ALS patients and healthy controls (HCs). Paired t-tests were used for self-comparisons of electrophysiological variables at different time points of cultivation. Significance was defined as p < 0.05. Statistical analyses were performed using SPSS 23.0 and GraphPad Prism 8.3.

Given the limited number of donor lines, statistical analyses were performed at the cellular level to capture functional heterogeneity within each line. We acknowledge that this approach does not account for donor-level effects, and results should be interpreted with this caveat. No correction for multiple comparisons was applied due to the exploratory nature of this study and the interrelated nature of electrophysiological parameters. Future studies with larger cohorts should employ appropriate corrections.

## 3 Results

### 3.1 ALS cases presentation

#### 3.1.1 Case 1

A 49-year-old male presented to our hospital with progressive limb weakness and atrophy over 5 months. Weakness gradually affected his right leg, left leg, and right hand. The patient reported no paresthesia or numbness. His medical history was unremarkable. The patient’s mother was diagnosed with ALS at age 56 and passed away 2 years later. Physical examination revealed muscle strength in right thumb abduction and finger extension at 4 on the Medical Research Council (MRC) scale. Severe weakness and atrophy were noted in both legs (2 MRC in the right leg and 3 MRC in the left leg). Tendon reflexes were normal in the upper limbs but reduced in the lower limbs. Bilateral Babinski signs were negative. ALSFRS-R score at enrollment was 38. Needle electromyography (EMG) revealed widespread neurogenic damage in muscles across the bulbar, cervical, thoracic, and lumbosacral regions. Whole-exome sequencing (WES) identified a c.260A>G (p.Asn87Ser) pathogenic mutation in the SOD1 gene. The patient became bedridden by October 2024 and required invasive respiratory support starting in December 2024. The time from symptom onset to the need for invasive respiratory support was 22 months.

#### 3.1.2 Case 2

A 32-year-old female visited our clinic due to progressive limb weakness and atrophy for 8 months. Weakness gradually involved her left leg, left hand and right hand. No paresthesia or numbness was reported. Past history and family history were unremarkable. Physical examination revealed weakness of left shoulder abduction, elbow flexion and extension (4 MRC level), left thumb abduction and finger extension (4- MRC level), right shoulder abduction, elbow flexion and extension (3 MRC level), right thumb abduction and finger extension (0 MRC level). Muscle strengths of left hip flexion, knee flexion, knee extension, ankle dorsiflexion and plantar flexion were 3, 3, 5, 0 and 0 MRC levels. For the right side, there were 4, 4, 5, 3 and 3 MRC levels. Mild atrophy was found in all limbs. Tendon reflexes were normal and bilateral Babinski signs were negative. ALSFRS-R score at enrollment was 32. Needle EMG reported widespread neurogenic damage involving muscles in bulbar, cervical, thoracic and lumbosacral regions. No pathological gene mutation was found by WES. The patient manifested dyspnea in March 2024 and died of respiratory failure in July 2024. The survival time from onset was around 20 months.

### 3.2 Characteristics and dynamic changes of motor neuron excitability

We observed four distinct evoked firing patterns in iPSC-derived motor neurons (MNs), including repetitive, adaptive, single, and no action potentials (APs) (Figure 1b), consistent with prior studies (Akçimen et al., 2023). Repetitive APs were observed only in 2–4 weeks-differentiated MNs with mutant SOD1 (2, 7.41%) (Figure 1c). During the 4–7 week period, mature iPSC-MNs from all groups exhibited four distinct firing patterns. Compared to immature MNs at 2–4 weeks, MNs displaying repetitive firing at 4 weeks showed hyperpolarized resting membrane potentials and increased AP peak amplitudes (Supplementary Table 1). There were no significant differences in the percentages of cells showing repetitive firing among MNs derived from healthy controls (HCs) (6, 17.14%), SOD1 (6, 21.43%), and sporadic ALS (sALS) (7, 16.28%) during the 4–7 week period (p > 0.05) (Figure 1d). During the 7–9 week period, the percentages of MNs showing repetitive firing patterns derived from mutant SOD1 (7, 30.44%) and sALS (8, 21.62%) were lower compared to those from HCs (7, 43.75%), but no statistical significance was observed (p > 0.05) (Figure 1e).

**FIGURE 2:**
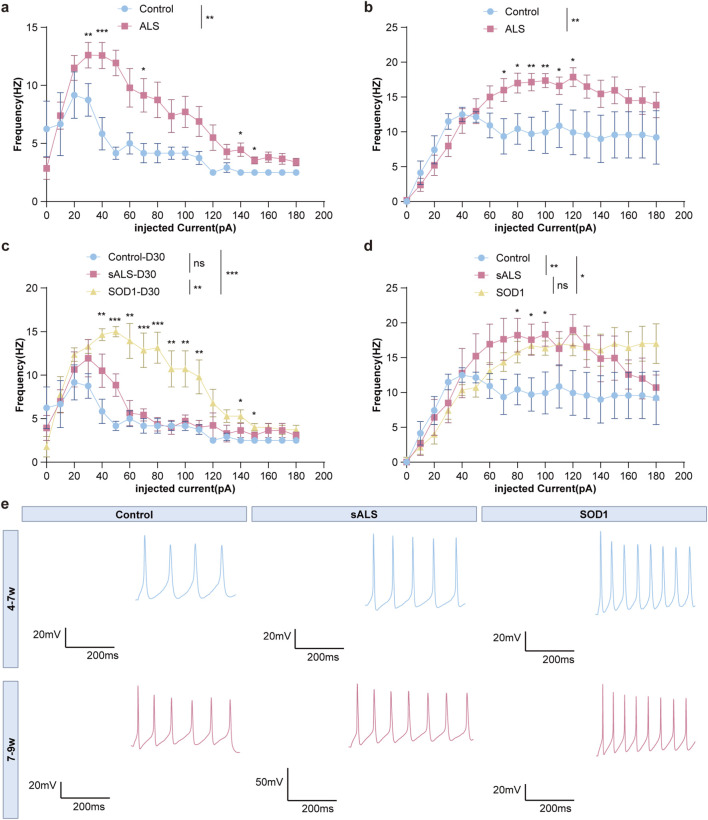
Frequency-Injected current relationships generated from repetitively firing iPSC-MNs cultured for different time periods; representative AP firing traces at 50 pA current injection. **(a,c)** 4–7 weeks, **(b,d)** 7–9 weeks. Mean ± s.e.m values were plotted in the graph. ns no significance, *p < 0.05, **p < 0.01, ***p < 0.001. **(e)** Repetitive AP traces taken from current clamp recordings in iPSC-derived MNs from control, sALS and SOD1 lines.

As presented in Figure 2, we compared the firing frequencies of MNs, which suggested that ALS patient-derived MNs exhibited significantly higher firing rates compared to HCs at both 4–7 weeks (p = 0.004) and 7–9 weeks (p = 0.009) (Figures 2a,b). Further analyses revealed that SOD1-derived MNs showed significantly higher firing frequencies than sALS (p = 0.009) and HCs (p < 0.001) in 4–7 weeks (Figure 2c). In 7–9 weeks, it remained significant between SOD1 and HC-derived MNs (p = 0.015), but became insignificant between SOD1 and sALS (p = 0.855) (Figure 2d). MNs derived from sALS showed a non-significant trend toward increased firing frequency in 4–7 weeks (p = 0.170), which became statistically significant by 7–9 weeks (p = 0.008) (Figures 2c,d).

**FIGURE 3:**
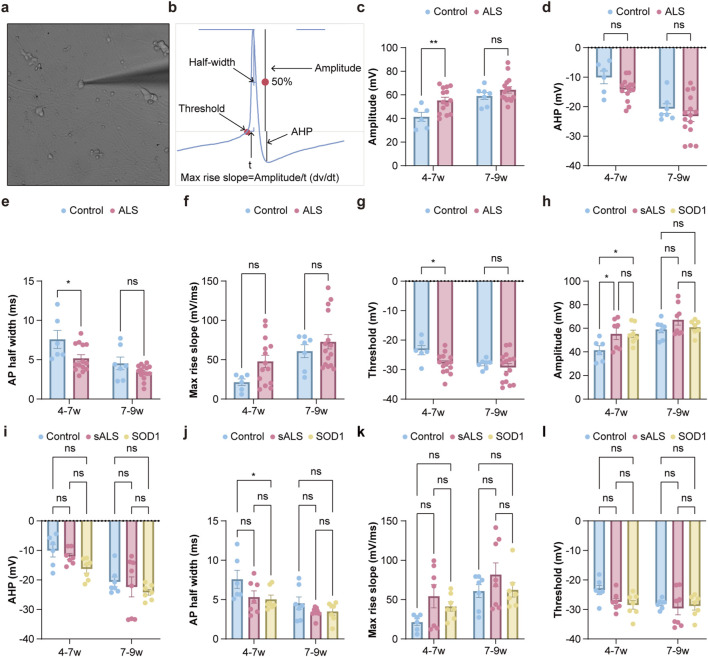
Comparisons in the characteristics of AP waveforms between HC and ALS iPSC-derived MNs cultured for different time periods. Whole-cell patch-clamp recordings of iPSC-derived MNs from control subjects and ALS patients at 4–7 weeks and 7–9 weeks. **(a)** Bright field images of iPSC-derived MNs in presence of the intracellular pipette for patch-clamp recordings. **(b)** APs of MNs and relevant indexes. **(c–g)** ALS vs. HC, **(h–I)** subgroup analyses. Quantification of AP amplitude, AHP, AP half-width, max rise slope, threshold. Data are presented as the Mean ± s.e.m; ns no significance, *p < 0.05, **p < 0.01, ***p < 0.001.

Further analysis of AP waveforms revealed significantly higher peak amplitude (54.75 ± 10.33 vs. 41.42 ± 9.21 mV, p = 0.008), lower half-width (4.85 ± 2.07 vs. 7.58 ± 2.80 ms, p = 0.023), and threshold (−28.69 ± 3.58 vs. −25.57 ± 3.91 mV, p = 0.042) in MNs derived from ALS patients compared to HCs at 4–7 weeks. Comparisons in AHP (p = 0.081) and maximum rise slope (p = 0.179) showed no significant differences. At the 7–9 week period, all AP waveform parameters were comparable between ALS and HC-derived MNs (Figures 3c–g). Subgroup analyses indicated that at 4–7 weeks, SOD1-derived MNs exhibited significantly higher amplitude (54.24 ± 8.46 vs. 41.42 ± 9.21 mV, p = 0.019) and lower half-width (4.45 ± 2.11 vs. 7.58 ± 2.80 ms, p = 0.034) compared to HCs. (Figures 3h,j) MNs derived from sALS only exhibited higher amplitude of AP (55.33 ± 12.83 vs. 41.42 ± 9.21 mV, p = 0.049) compared to HCs at 4–7 weeks (Figure 3h). Comparisons in AHP (p = 0.778), maximum rise slope (p = 0.7075) and threshold (p = 0.3807) showed no significant differences (Figures 3i,k,l). As shown in Supplementary Figure 4, longitudinal comparisons within the same line of MNs revealed that peak amplitude (p = 0.007) and maximum rise slope (p = 0.038) of HC-derived MNs exhibited increasing trends with prolonged culture duration, while AHP (p = 0.003) and half-width (p = 0.007) showed decreasing trends over time. Similar directional changes were observed in ALS-derived MNs, but only reductions in AHP (p < 0.001) and half-width (p = 0.021) reached statistical significance. Furthermore, AHP was significantly lower at 4–7 weeks compared to 7–9 weeks in both sALS (p = 0.003) and SOD1 (p = 0.003) derived MNs. No other parameters exhibited statistically significant differences between the two time points.

### 3.3 Characteristics and dynamic changes of motor neuron morphology

**FIGURE 4:**
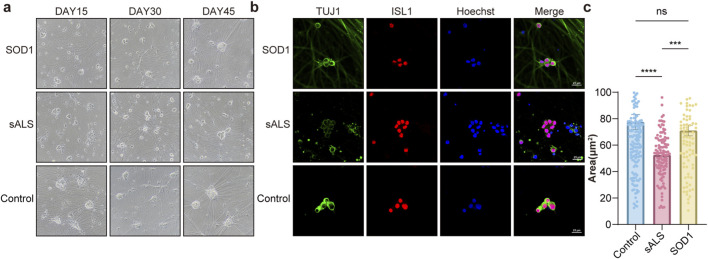
Comparisons in soma size of MNs between HCs and ALS. **(a)** Light microscopic image of MNs. **(b)** Immunofluorescence staining of MNs at Day 30. Representative images showing soma of control, sALS, and SOD1 MNs (scale bar = 25 μm). **(c)** Quantification of ISL1 positive MN soma size. Control: n = 156; sALS: n = 114; SOD1: n = 90. N = number of recorded MNs from three independent experiments. ns no significance, ***p < 0.001, ****p < 0.0001.

After differentiating iPSCs into mature motor neurons (MNs), we maintained the cultures and monitored changes in neuronal morphology over time (Figures 4a,b). Quantitative analysis using ImageJ software at Day 30 post-differentiation revealed that the soma size of MNs derived from sALS (52.66 ± 17.33 μm) was significantly smaller than that of MNs derived from healthy controls (HCs) (77.57 ± 71.07 μm, p < 0.001) and SOD1 (71.11 ± 38.15 μm, p < 0.001). Notably, no significant difference in soma size was observed between MNs derived from HCs and SOD1 (p = 0.428) (Figure 4c). Unfortunately, upon continued culture to Day 60, the soma of MNs in all groups aggregated into clusters, preventing further measurements and analysis.

**FIGURE 5:**
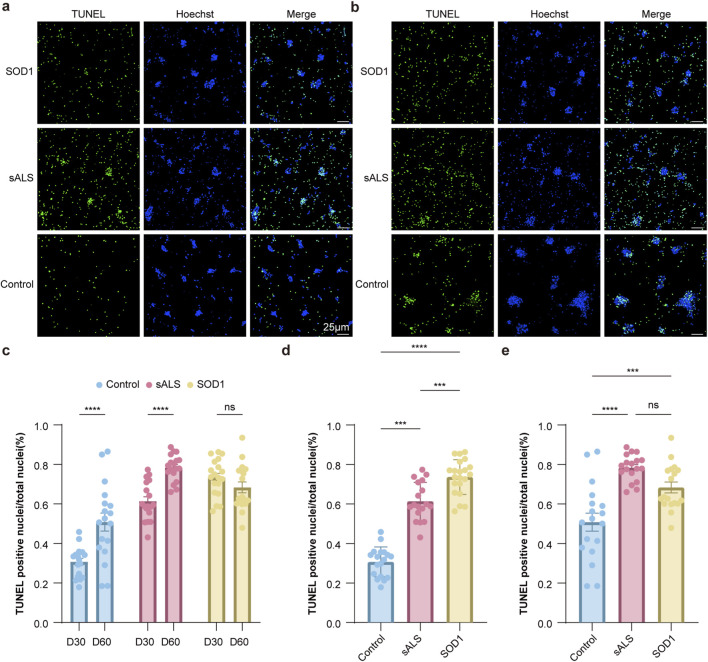
iPSC-derived MNs from sALS and SOD1 ALS patients exhibit survival differences relative to healthy controls. **(a)** TUNEL positive nuclei of neuronal cultures without glia at Day 30 (n = 3,m > 1000). **(b)** TUNEL positive nuclei of neuronal cultures without glia at Day 60 (n = 3,m > 1000) (scale bar = 25 μm). **(c)** Longitudinal comparisons, **(d)** Comparisons between ALS and HCs at Day 30, **(e)** Comparisons between ALS and HCs at Day 60. n = experiment, m = cell number. Data are presented as the Mean ± s.e.m; ns no significance, ***p < 0.001, ****p < 0.0001.

As shown in Figure 5, the apoptotic rate of sALS (Day 30: 61.37% ± 9.63%, p < 0.001; Day 60: 78.41% ± 6.63%) and SOD1 (Day 30: 73.69% ± 8.81%, p < 0.001; Day 60: 60.37% ± 11.53%, p < 0.001) derived MNs was significantly higher than that of HCs (Day 30: 30.72% ± 7.57%; Day 60: 50.85% ± 19.36%) at both Day 30 and Day 60. At Day 30, the apoptotic rate of SOD1-derived MNs was significantly higher than that of sALS (p = 0.002), but was comparable at Day 60 (p = 0.078). Longitudinal comparisons revealed that the apoptotic rate of sALS-derived MNs exhibited an increasing trend with prolonged culture time (p < 0.001). SOD1-derived MNs consistently exhibit a high apoptotic rate and no significant change occurred longitudinally. The apoptotic rate of HC-derived MNs also increased significantly from Day 30 to Day 60 (p = 0.022, as shown in Figure 5c), though remaining lower than ALS lines at both time points.

## 4 Discussion

It is widely accepted that the onset of ALS results from a combination of genetic and environmental factors (Akçimen et al., 2023; Motataianu et al., 2022). For ALS patients with well-defined causative genes, the mechanisms are relatively clear. However, for those without identified causative genes, the pathogenesis may be linked to genetic susceptibility, and the specific mechanisms remain to be elucidated (Akçimen et al., 2023). Although multiple hypotheses exist, the environmental factors contributing to ALS are not yet fully understood. These factors typically include the intensity and duration of exposure to environmental risks. In this study, motor neurons derived from iPSCs of SOD1-related ALS, sporadic ALS, and healthy controls (HCs) were cultured in the same environment, thus eliminating environmental factors. By removing these environmental variables, the study of iPSC-derived motor neurons primarily reflects the genetic background underlying ALS pathogenesis (Du et al., 2023). Our study found that motor neurons derived from both SOD1 gene related ALS and sporadic ALS, showed increased excitability and higher degrees of apoptosis during culture process compared with healthy individuals. In previous studies about iPSC derived motor neurons of ALS mainly focused on ALS patients with well-defined pathogenic genes (Ma et al., 2025). Our study shows that motor neurons derived from sporadic ALS patients also have increased excitability and can be used as models for sporadic ALS studies.

Our study showed that the excitability and apoptosis of motor neurons differentiated from iPSCs of ALS patients exhibited dynamic changes, with differences observed between SOD1 gene mutation-related ALS and sporadic ALS. The excitability of motor neurons derived from patients with SOD1 gene mutations increased earlier, and the percentage of apoptosis was higher than that of motor neurons from sporadic ALS patients at the early stages of culture. However, at the later stages of culture (7–9 weeks), the excitability of motor neurons from sporadic ALS patients increased significantly, resulting in no significant difference in excitability or apoptosis between the two types of motor neurons. The differences in the dynamic changes in excitability and apoptosis between the two types of motor neurons during culture are likely related to their genetic differences. In motor neurons with SOD1 gene mutations, the abnormal protein levels caused by these mutations have a more severe impact on motor neuron function. In contrast, sporadic ALS-derived motor neurons, despite the absence of a clear pathogenic gene, still exhibit genetic susceptibility, which leads to increased motor neuron excitability and apoptosis through specific mechanisms. Although the degree of abnormality is relatively mild initially, with prolonged culture time, the severity of these changes can reach levels similar to those observed in SOD1 gene mutation-related ALS. Our data show a temporal association between early hyperexcitability and subsequent apoptosis, suggesting a potential mechanistic link. While causality cannot be inferred from these correlative data, the parallel dynamics support further investigation into whether excitotoxicity contributes to MN loss in ALS iPSC models. The observed early hyperexcitability may contribute to subsequent MN degeneration through increased metabolic demand, calcium overload, and excitotoxic stress (Hammer, 2025). Persistent elevated firing could lead to mitochondrial dysfunction and activation of apoptotic pathways, linking the electrophysiological and viability phenotypes observed here (Arnold et al., 2024). This aligns with proposed mechanisms in SOD1 models and underscores excitability modulation as a potential therapeutic target not only for familial but also sporadic ALS (Wong and Venkatachalam, 2019). These data suggest that early dysfunction or loss of ion channels may serve as a convergent point contributing to the initiation of downstream degenerative pathways, ultimately leading to motor neuron (MN) loss in ALS.

Motor neuron (MN) excitability dysregulation is a precursor to synaptic failure, axonal degeneration, and cell death—driven by remodeling of core electrical properties: action potential (AP) threshold, AP amplitude, and AP half-width. However, sALS and SOD1-ALS exhibit divergent patterns of electrical property change (Elbasiouny, 2022). SOD1-ALS is defined by monophasic, early-onset MN hyperexcitability driven by coordinated remodeling of active and passive electrical properties, with the most impactful changes in frequency, AP threshold and half-width (Wainger et al., 2014). The primary electrical hallmark of early SOD1-ALS MNs is a hyperpolarized AP threshold,5–10 mV more negative compared to controls, which directly increases excitability by enabling APs in response to subthreshold inputs. And the AP half-width is narrowed, a change that amplifies hyperexcitability-induced damage in SOD1-ALS (Huang et al., 2021). sALS-derived MNs exhibits a more heterogeneous, stage-dependent excitability phenotype: early subtle hyperexcitability to late-stage robust hyperexcitability. In late stage (7–9 weeks), sALS-derived shows higher firing frequencies and narrow half-width compared to the early stage (4–7 weeks). (Supplementary Figure 3h). TDP-43 mislocalization indirectly disrupts ion channel transcription/splicing (Nav1.6, Kv2.1), while reactive astrocytes and microglia amplify electrical dysfunction (Djukic et al., 2025). This dual driver leads to slower, more variable electrical remodeling and stage-dependent excitability shifts. These differences reflect their unique molecular drivers—MN-intrinsic SOD1 ion channel interactions in SOD1-ALS, and TDP-43 pathology + non-cell-autonomous astrocyte effects in sALS (Geevasinga et al., 2016; Scekic-Zahirovic et al., 2024).

The spontaneous increase in apoptosis over time (Day 30 to Day 60) in healthy control MN cultures is a multifactorial phenomenon that does not stem from a single cause but reflects the integrated effects of culture-related microenvironmental stress, intrinsic neuronal maturation programming, and physiological baseline turnover of neural cells (Xu et al., 2004). Subsequent work have consistently shown that neuronal survival *in vitro* is highly dependent on optimized, neurocentric media formulations, with decline over time being a standard challenge (Smirnova et al., 1998; Pitrez et al., 2024). Thiry L et al. (Thiry et al., 2022)and others have noted that iPSC-derived neurons often exhibit hallmarks of fetal-stage cells, including ongoing developmental processes like natural apoptosis. Studies on human iPSC-derived neural cultures have shown that healthy control neurons (including MNs) exhibit a 2–3-fold increase in ROS levels and caspase-3 activity from Day 30 to Day 60, directly correlating with elevated apoptotic rates (Yu et al., 2025). The study on C9ORF72 ALS/FTD organoid models (Szebényi et al., 2021) shows that healthy control ALI-COs (containing MN-like deep layer neurons) exhibit a mild increase in apoptosis from Day 30–60, which is attributed to culture-related hypoxic stress and developmental maturation—this apoptosis is not associated with DNA damage or poly (GA) accumulation and is reversed by improving air-liquid interface culture conditions.

ALS usually develops in old age, but in this study, we selected a 32-year-old young adult with ALS for iPSC differentiated motor neuron study. The patient developed ALS at an early age, but WES failed to detect the disease-causing gene. The failure to detect the pathogenic gene may be due to the fact that the patient has a certain gene, which is not defined as disease causing gene; it may also be that there is no pathogenic gene, but like other sporadic ALS, there is a genetic susceptibility factor, which through some mechanism has a stronger influence on motor neuron excitability. This leads to an earlier age of onset. The age of onset in patients with SOD1 gene mutation was 49 years old, which was later than that in sporadic patients, but the degree of abnormal motor neuron excitability was more obvious than that in sporadic patients. Theoretically, the age of onset in patients with SOD1 gene mutation should be earlier (Opie-Martin et al., 2024). This discrepancy may be due to stronger environmental factors involved in sporadic ALS. The onset of ALS in China generally occurs earlier than in Europe and the United States, suggesting that environmental factors may play a significant role (Shen et al., 2024).

Although live immunolabeling was not used during recordings, *post hoc* staining confirmed high MN purity. Nevertheless, we cannot exclude the possibility that a small proportion of recorded cells were interneurons, which may contribute to variability in electrophysiological measures. Furthermore, our culture system contained a mixed population of neurons and glia. Although not quantified here, the presence of astrocytes—which are known to contribute to MN toxicity in ALS through inflammatory signaling and impaired support functions—may have influenced MN excitability and survival. In previous studies, GFAP + astrocytes exist as an essential supporting cell population with a stable and low basal proportion in most co-culture systems (Szebényi et al., 2021). In the process of inducing pluripotent stem cells to differentiate into MNs and maturing *in vitro*, the proportion of GFAP + astrocytes shows a slow and gradual increase trend: in the early stage of differentiation (0–30 days *in vitro*), the culture is dominated by neural progenitors and immature MNs, with almost no GFAP + astrocytes; in the middle stage (30–100 days *in vitro*), astrocytes begin to differentiate and mature, and the GFAP + proportion rises to 5%–10%; in the late mature stage (100 days *in vitro* and above), it stabilizes at the physiological ratio of 10%–20% (Cervetto et al., 2023). The C9ORF72 ALI-CO model clearly shows that at 150 days *in vitro*, the GFAP + astrocyte proportion in mutant organoids is consistent with that in healthy and isogenic corrected control groups, and single-cell RNA-seq further confirms that the cell type composition ratio of glia and neurons is preserved (Szebényi et al., 2021). Similar results have been obtained in SOD1 mutant mouse primary MN-astrocyte co-cultures: the number of GFAP + astrocytes does not increase, but the intensity of GFAP immunofluorescence is significantly upregulated (Wong and Venkatachalam, 2019). The C9ORF72 ALI-CO model is the first to reveal that GFAP + astrocytes in disease MN cultures exhibit early and specific endoplasmic reticulum (ER) stress: the ratio of phospho-EIF2α/EIF2α is increased by 1.79 times, the stress granule marker PABP1 is upregulated by 2.67 times, and the autophagy signaling protein P62 is increased by 1.86 times compared with controls (Szebényi et al., 2021). In SOD1 mutant astrocyte-MN co-cultures, activated GFAP + astrocytes also show unfolded protein response (UPR) activation, and the secretion of misfolded proteins is increased, which directly induces MN proteostasis disorder and synaptic damage (Smith et al., 2020).

As a preliminary investigation into the excitability of motor neurons derived from iPSCs of patients with SOD1 gene mutations and sporadic ALS, the small sample size limits the representativeness of the findings. Future studies should aim to expand the sample size to better understand the excitability characteristics of motor neurons derived from both sporadic ALS and gene mutation-related ALS, as well as the underlying mechanisms leading to abnormal excitability. No correction for multiple comparisons was applied due to the exploratory nature of this study and the interrelated nature of electrophysiological parameters. Future studies with larger cohorts should employ appropriate corrections.

## 5 Conclusion

In summary, motor neurons derived from both patients with SOD1 gene mutations and sporadic ALS exhibited increased excitability during the early stages of culture. The excitability of motor neurons from SOD1 gene mutation patients increased earlier, and over time, the excitability of motor neurons from both ALS groups became more consistent. The apoptosis rates of motor neurons derived from both SOD1-related ALS and sporadic ALS were significantly higher than those of healthy controls, with the apoptosis in SOD1-related ALS motor neurons being more pronounced in the early stages. Therefore, motor neurons derived from iPSCs of both sporadic and SOD1 gene mutation-related ALS can serve as valuable cell models for ALS. Analyzing the dynamic changes in their excitability will provide insights into ALS pathogenesis and could contribute to future drug development, especially for sporadic ALS. Future studies with larger cohorts are necessary to validate these dynamic changes and to explore subtype-specific mechanisms.

## Data Availability

The raw data supporting the conclusions of this article will be made available by the authors, without undue reservation.

## Supplementary material

SUPPLEMENTARY FIGURE 1 Generation of iPSC lines of sALS and SOD1 ALS patients and HC. **(a)** G-banding karyotype analysis showing normal chromosome number with no deletions or duplications, and no structural abnormalities. **(b)** Light microscopic image of iPSCs. **(c)** Cell Pluripotency Detection showing Alkaline Phosphatase staining positive. **(d)** PCR electrophoresis results were negative, with no *mycoplasma* contamination detected.

SUPPLEMENTARY FIGURE 2 Immunofluorescence images of iPSC lines expressing markers Oct 4, Nanog, Sox2, SSEA4. (scale bar = 200 μm).

SUPPLEMENTARY FIGURE 3 Generation of iPSC-derived MNs. Neurons were immunostained for TUJ1 (green) and HB9 (red) at Day 5. Nuclei were stained with Hoechst (blue). **(a-d)** Quantitation of ISL1 positive and TUJ1 positive MNs at Day 30.Neurons were immunostained for TUJ1 (green) and ISL1 (red). (n = 3, scale bar = 100 μm) **(e)** Schematic illustrating differentiation of iPSCs into early MN ns no significance.

SUPPLEMENTARY FIGURE 4 Longitudinal comparisons in the characteristics of AP waveforms of HC and ALS iPSC-derived MNs cultured for different time periods. **(a-e)** ALS vs. HC, **(f-j)** sALS vs. SOD1 analyses. ns no significance, *p < 0.05, **p < 0.01, ***p < 0.001.
