# Supplementary material for: Dynamic changes in excitability and viability of sporadic and SOD1-related amyotrophic lateral sclerosis iPSC-derived motor neurons
Source: Front Cell Dev Biol. 2026 Mar 30;14:1755814. doi: 10.3389/fcell.2026.1755814 (PMC13070925; doi:10.3389/fcell.2026.1755814)
Supplement: Supplementary file 1 [file Table1.docx]

**Supplementary Table 1.** **Passive membrane properties in iPSC-derived motor neurons from sporadic ALS, SOD1 and HC at different culture stages.**

|  | SOD1 | sALS | Control | One-way ANOVA |
| --- | --- | --- | --- | --- |
| Cm (pF) |  |  |  |  |
| ≤14d | 13.93±4.87 | 17.47±6.82 | 15.51±9.54 | 0.512 |
| 14-49d | 26.65±10.64 | 25.69±18.37 | 23.95±15.31 | 0.674 |
| ≥50d | 34.81±14.74 | 19.63±5.42 | 24.38±4.73 | **<0.001** |
| Rn (MΩ) |  |  |  |  |
| ≤14d | 591.55±174.25 | 566.66±188.20 | 358.65±96.01 | 0.084 |
| 14-49d | 500.32±181.62 | 629.79±538.03 | 512.04±187.94 | 0.124 |
| ≥50d | 472.80±212.86 | 425.77±100.99 | 639.00±295.49 | **0.003** |
| RMP (mV) |  |  |  |  |
| ≤14d | -31.20±6.66 | -42.44±5.58 | -27.03±11.09 | **0.002** |
| 14-49d | -40.52±8.24 | -44.00±10.48 | -38.63±8.59 | **0.011** |
| ≥50d | -56.06±5.94 | -46.78±6.18 | -49.36±5.28 | **<0.001** |

Abbreviations: ALS amyotrophic lateral sclerosis, CM capacitance of membrane, HC healthy controls, iPSC induced pluripotent stem cells, RMP resting membrane potential, Rn membrane resistance.

Note: Data are presented as the Mean±s.e.m. P-values from one-way ANOVA tests. P<0.05 was considered significance, which has been bold.
