# Supplementary figures and images for: Dynamic changes in excitability and viability of sporadic and SOD1-related amyotrophic lateral sclerosis iPSC-derived motor neurons
Source: Front Cell Dev Biol. 2026 Mar 30;14:1755814. doi: 10.3389/fcell.2026.1755814 (PMC13070925; doi:10.3389/fcell.2026.1755814)

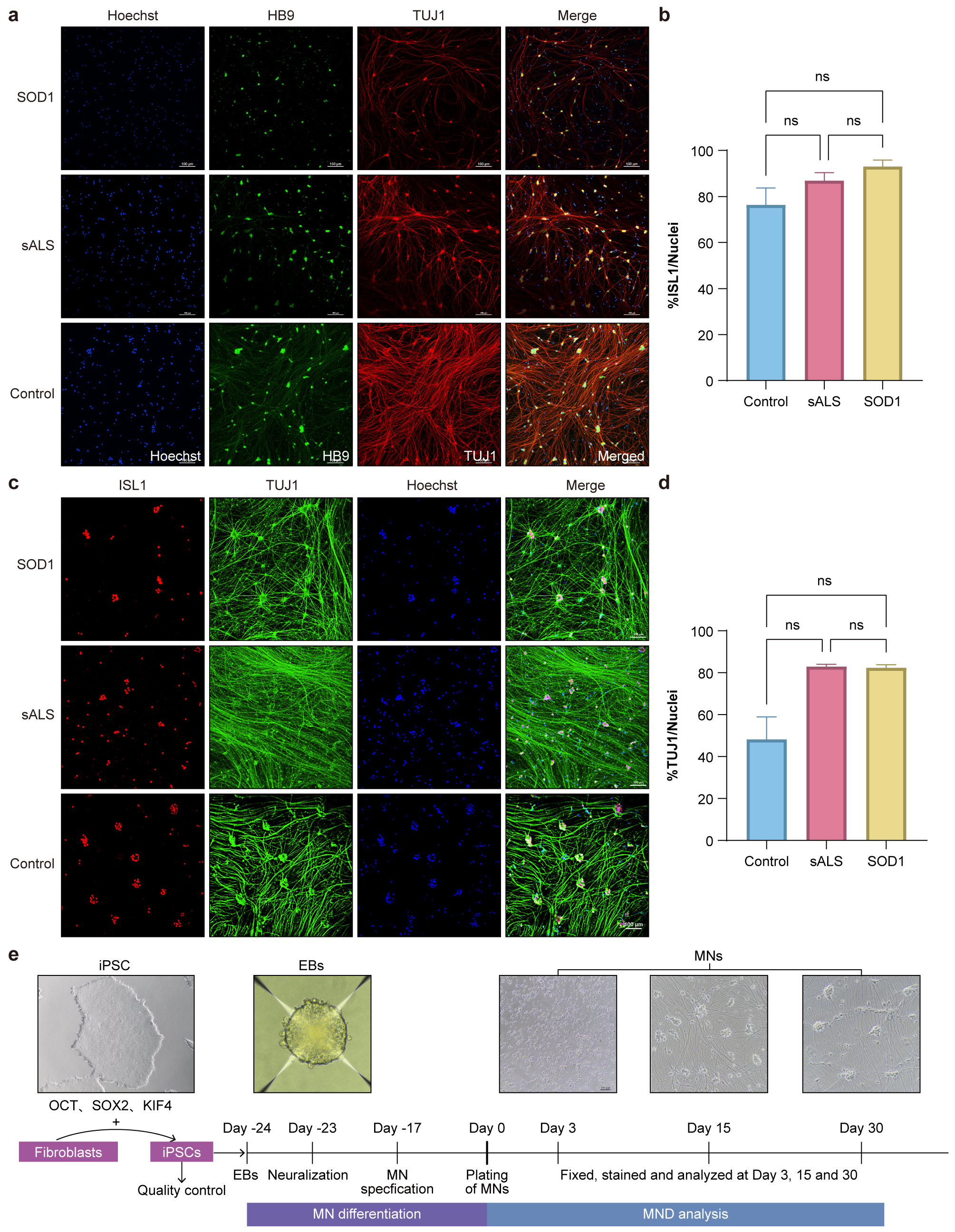

Supplement: Supplementary file 2 [file Image3.tif]

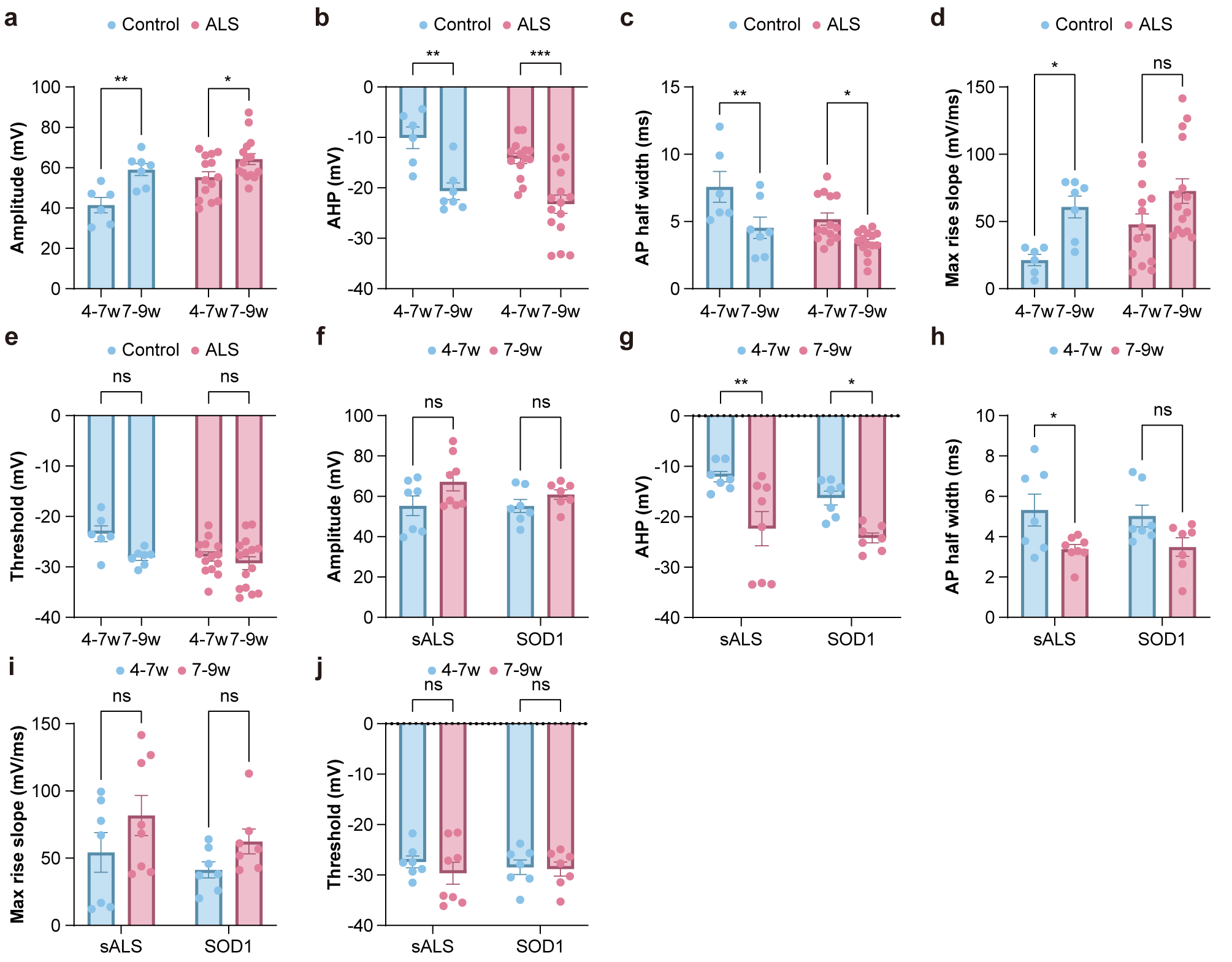

Supplement: Supplementary file 3 [file Image4.tif]

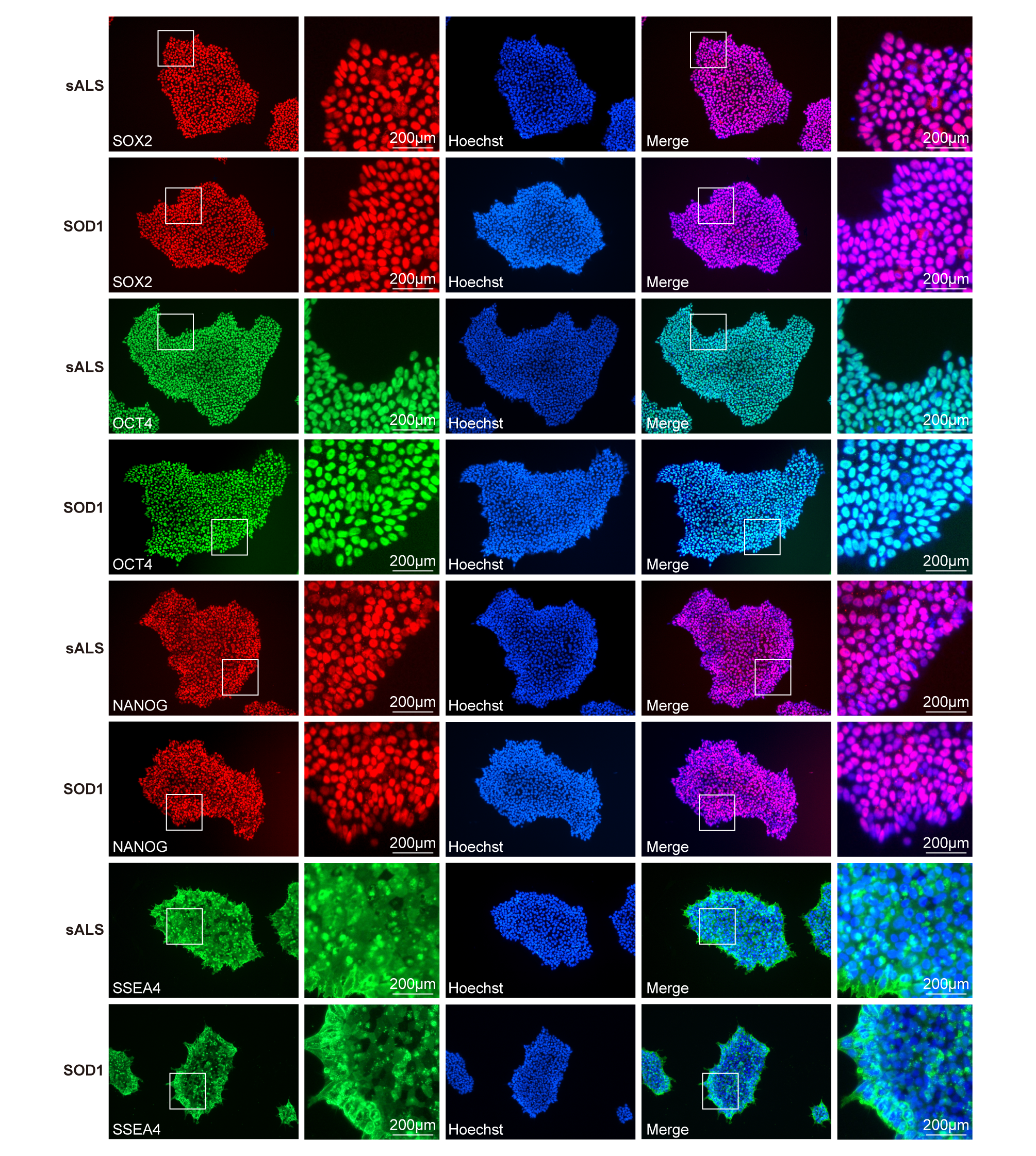

Supplement: Supplementary file 4 [file Image2.tif]

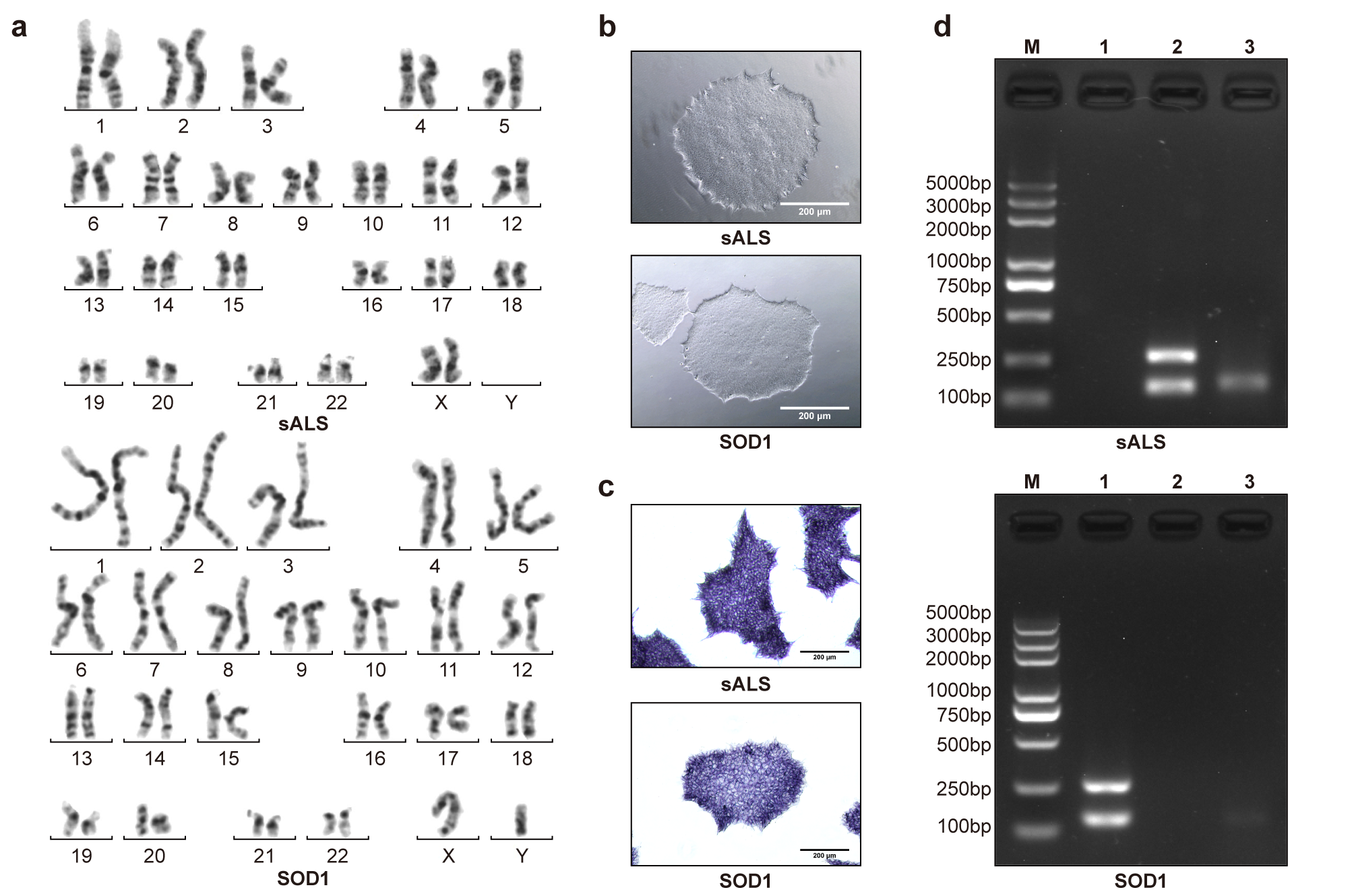

Supplement: Supplementary file 5 [file Image1.tif]
